# Defining regulatory and phosphoinositide-binding sites in the human WIPI-1 β-propeller responsible for autophagosomal membrane localization downstream of mTORC1 inhibition

**DOI:** 10.1186/1750-2187-7-16

**Published:** 2012-10-22

**Authors:** Anja Gaugel, Daniela Bakula, Anneliese Hoffmann, Tassula Proikas-Cezanne

**Affiliations:** 1 From the Autophagy Laboratory, Department of Molecular Biology, Interfaculty Institute of Cell Biology, Eberhard Karls University Tuebingen, Auf der Morgenstelle 15, 72076, Tuebingen, Germany

**Keywords:** Autophagosome, Autophagy, Atg12, Atg18, Phagophore, PtdIns3KC3, PtdIns(3)P, PtdIns(3,5)P_2_, WIPI-1, YM201636

## Abstract

**Background:**

Autophagy is a cytoprotective, lysosomal degradation system regulated upon induced phosphatidylinositol 3-phosphate (PtdIns(3)P) generation by phosphatidylinositol 3-kinase class III (PtdIns3KC3) downstream of mTORC1 inhibition. The human PtdIns(3)P-binding β-propeller protein WIPI-1 accumulates at the initiation site for autophagosome formation (phagophore), functions upstream of the Atg12 and LC3 conjugation systems, and localizes at both the inner and outer membrane of generated autophagosomes. In addition, to a minor degree WIPI-1 also binds PtdIns(3,5)P_2_. By homology modelling we earlier identified 24 evolutionarily highly conserved amino acids that cluster at two opposite sites of the open Velcro arranged WIPI-1 β-propeller.

**Results:**

By alanine scanning mutagenesis of 24 conserved residues in human WIPI-1 we define the PtdIns-binding site of human WIPI-1 to critically include S203, S205, G208, T209, R212, R226, R227, G228, S251, T255, H257. These amino acids confer PtdIns(3)P or PtdIns(3,5)P_2_ binding. In general, WIPI-1 mutants unable to bind PtdIns(3)P/PtdIns(3,5)P_2_ lost their potential to localize at autophagosomal membranes, but WIPI-1 mutants that retained PtdIns(3)P/PtdIns(3,5)P_2_ binding localized at Atg12-positive phagophores upon mTORC1 inhibition. Both, downregulation of mTOR by siRNA or cellular PtdIns(3)P elevation upon PIKfyve inhibition by YM201636 significantly increased the localization of WIPI-1 at autophagosomal membranes. Further, we identified regulatory amino acids that influence the membrane recruitment of WIPI-1. Exceptional, WIPI-1 R110A localization at Atg12-positive membranes was independent of autophagy stimulation and insensitive to wortmannin. R112A and H185A mutants were unable to bind PtdIns(3)P/PtdIns(3,5)P_2_ but localized at autophagosomal membranes, although in a significant reduced number of cells when compared to wild-type WIPI-1.

**Conclusions:**

We identified amino acids of the WIPI-1 β-propeller that confer PtdIns(3)P or PtdIns(3,5)P_2_ binding (S203, S205, G208, T209, R212, R226, R227, G228, S251, T255, H257), and that regulate the localization at autophagosomal membranes (R110, R112, H185) downstream of mTORC1 inhibition.

## Background

Macroautophagy (hereafter referred to as autophagy) is defined as a stochastic lysosomal bulk degradation pathway for cytoplasmic material, and characterized by membrane rearrangements from autophagosome formation to fusion events with the lysosomal compartment. In addition, autophagy selectively targets the degradation of protein aggregates, damaged organelles and invading pathogens. Stochastic and selective autophagy control both turnover and clearance of the cytoplasm, thereby critically contributing to eukaryotic cell homeostasis (for review see e.g. [1-4]).

The autophagosomal membrane is of as yet uncertain origin [5], but recent reports provide evidence that multiple membrane systems, such as the endoplasmic reticulum [6,7] or the plasma membrane [8] contribute to autophagosome formation from initial template membranes (phagophore). Phagophores are proposed to elongate to double-membrane autophagosomes by receiving membrane input from the endocytic compartment, critically involving Atg9-positive vesicles [9]. Autophagosomes sequester the cytoplasmic cargo and communicate or fuse with the lysosomal compartment to permit final degradation [10].

The AMPK/mTORC1 sensing system for nutrient, energy and hormone level modulates the onset of autophagy via differential phosphorylation of the Ulk1-Atg13-FIP200 complex [11-15]. Within this control circuit, mTORC1 negatively regulates autophagy, first demonstrated by rapamycin-mediated induction of autophagy [16]. mTORC1 inhibition promotes the activation of phosphatidylinositol-3 kinase class III (PtdIns3KC3, Vps34) that generates phosphatidylinositol 3-phosphate (PtdIns(3)P) [17,18]. Generation of PtdIns(3)P is prerequisite for the formation of autophagosomes [19], first demonstrated by the employment wortmannin, an irreversible phosphatidylinositol 3-kinase inhibitor [20].

PtdIns3KC3 becomes engaged in the initiation of autophagy through complex formation with Beclin 1, p150 (Vps15) and Atg14L, the latter recruiting PtdIns3KC3 to the ER [21,22] where PtdIns(3)P-effectors subsequently contribute to the formation of autophagosomes [23-25]. PtdIns(3)P-binding proteins shown to be involved in the process of autophagy include Alfy [26] and DFCP1 [23,27] both of which bind PtdIns(3)P at phagophore precursors via their the FYVE-domain [28]. Factors belonging to the WD-repeat protein interacting with phosphoinositide (WIPI) family fold into seven-bladed β-propeller proteins with an open Velcro topology and bind PtdIns(3)P at the phagophore [29-31]. Human WIPI-1 and WIPI-2 evolved from the ancestral yeast Atg18 protein and share an essential function during autophagosome formation upstream of the Atg12 and LC3 conjugation systems, hence regulate LC3 conjugation to phosphatidylethanolamine (LC3-II) [24,29,32]. WIPI-1 localizes to both ER and PM, and WIPI-2 was found to localize close to the Golgi area and to the PM upon the induction of autophagy [33]. Further, both WIPI-1 and WIPI-2 were detected at the inner and outer autophagosomal membrane [33]. From this, WIPI-1 and WIPI-2 should function as PtdIns(3)P effectors essential for decoding the PtdIns(3)P signal downstream of PtdIns3KC3, thereby permitting the recruitment of downstream autophagy-related (Atg) proteins [34,35].

Our previous phylogenetic analyses identified 24 evolutionarily conserved amino acids specific to the 7-bladed WIPI beta-propeller protein family [29]. These conserved amino acids cluster at two proposed binding sites of the WIPI propeller: one less conserved across the top of propeller blades 1–3 and one highly conserved across the bottom of blades 4–7. We proposed that the conserved amino acid cluster across the bottom of blades 4–7 confers binding to phosphoinositides (PtdIns) [29], because two arginine residues (R226/R227) situated within were shown to be critical for PtdIns(3)P binding [36].

By alanine scanning mutagenesis of the 24 evolutionarily conserved amino acids of the WIPI family, we functionally define here the critical amino acids in human WIPI-1 responsible for PtdIns-binding and autophagosomal membrane recruitment upon the induction of autophagy.

## Results

**Figure 1:**
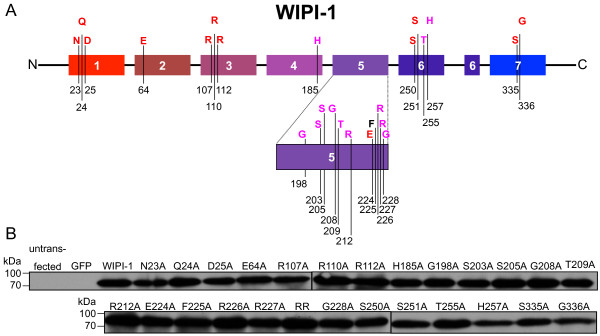
**Generation and transient expression of GFP-WIPI-1 mutants in human U2OS cells. (A)** Representation of the human WIPI-1 protein harbouring 7 WD repeats (coloured boxes 1–7) and evolutionarily conserved amino acids (homologous amino acids in red, invariant amino acids in purple, not conserved residue in black). **(B)** Transient over-expression and anti-GFP ECL analysis of GFP-tagged wild-type WIPI-1 (labelled WIPI-1) or generated mutant WIPI-1 variants (labelled N23A – G336) along with the GFP control and untransfected U2OS cells. The RR mutant carries alanine substitutions for both R226 and R227. Supplementary material provides an amino acid sequence alignment of wild-type and mutant WIPI-1 variants deduced from automated DNA sequencing upon site-directed mutagenesis (Additional file 1: Figure S1).

Site-directed alanine-screening mutagenesis of GFP-WIPI-1 (Table 1) was conducted to investigate the functional relationship of 24 conserved residues, unique to the WIPI protein family (Figure 1A, Additional file 1: Figure S1), with regard to both autophagosomal membrane localization (fluorescent puncta) and PtdIns-binding capabilities.

**Table 1:** Site-directed mutagenesis of human WIPI-1

**Mutant**	**Position**	**Mutation**	**PCR oligonucleotides (forward, reverse)**
GFP-N23A	N67-68	AA to GC	AGCTGCTTCTCTTTCGCCCAGGACTGCACATCC,
			GGATGTGCAGTCCTGGGCGAAAGAGAAGCAGCT
GFP-Q24A	N70-71	CA to GC	CTCTTTCAACGCGGACTGCACATCCCTAGCAA,
			TTGCTAGGGATGTGCAGTCCGCGTTGAAAGAG
GFP-D25A	N74	A to C	TCTTTCAACCAGGCCTGCACATCCCTAGCA,
			TGCTAGGGATGTGCAGGCCTGGTTGAAAGA
GFP-E64A	N191	A to C	GTCTACATCGCGGCGCGCCTCTTCTCC,
			GGAGAAGAGGCGCGCCGCGATGTAGAC
GFP-R107A	N319-320	AG to GC	CAACATCTTGTCCATAGCGCTGAACCGGCAAAGGC,
			GCCTTTGCCGGTTCAGCGCTATGGACAAGATGTTG
GFP-R110A	N328-329	CG to GC	TCCATAAGGCTGAACGCGCAAAGGCTGCTGGTT,
			AACCAGCAGCCTTTGCGCGTTCAGCCTTATGGA
GFP-R112A	N334-335	AG to GC	GCTGAACCGGCAAGCGCTGCTGGTTTGCC,
			GGCAAACCAGCAGCGCTTGCCGGTTCAGC
GFP-H185A	N553-554	CA to GC	TGCACTATTGCTGCCGCTGAGGGAACACTAGCTGCC,
			GGCAGCTAGTGTTCCCTCAGCGGCAGCAATAGTGCA
GFP-G198A	N593	G to C	CACCTTCAATGCCTCAGCCTCCAAACTAGCA,
			TGCTAGTTTGGAGGCTGAGGCATTGAAGGTG
GFP-S203A	N607-608	AG to GC	GGCTCCAAACTAGCAGCTGCGTCTGAAAAAGGC,
			GCCTTTTTCAGACGCAGCTGCTAGTTTGGAGCC
GFP-S205A	N613	T to G	CTAGCAAGTGCGGCTGAAAAAGGCACAGTC,
			GACTGTGCCTTTTTCAGCCGCACTTGCTAG
GFP-G208A	N623	G to C	AGTGCGTCTGAAAAAGCCACAGTCATCCGG,
			CCGGATGACTGTGGCTTTTTCAGACGCACT
GFP-T209A	N625	A to G	TCTGAAAAAGGCGCAGTCATCCGGGTG,
			CACCCGGATGACTGCGCCTTTTTCAGA
GFP-R212A	N634-635	CG to GC	GGCACAGTCATCGCGGTGTTCTCTGTCCC,
			GGGACAGAGAACACCGCGATGACTGTGCC
GFP-E224A	N671	A to C	GGCAAAAGCTCTATGCGTTCCGGAGAGG,
			CCTCTCCGGAACGCATAGAGCTTTTGCC
GFP-F225A	N673-674	TT to GC	GCAAAAGCTCTATGAGGCCCGGAGAGGGATGAA,
			TTCATCCCTCTCCGGGCCTCATAGAGCTTTTGC
GFP-R226A	N676-677	CG to GC	CAAAAGCTCTATGAGTTCGCGAGAGGGATGAAAAGGTATG,
			CATACCTTTTCATCCCTCTCGCGAACTCATAGAGCTTTTG
GFP-R227A	N679-680	AG to GC	AAAAGCTCTATGAGTTCCGGGCAGGGATGAAAAGGTATGT,
			ACATACCTTTTCATCCCTGCCCGGAACTCATAGAGCTTTT
GFP-RR	N676-677	CG to GC	GGGCAAAAGCTCTATGAGTTCGCTGCAGGGATGAAAAGGTATGTGAC,
	N679-680	AG to GC	GTCACATACCTTTTCATCCCTGCAGCGAACTCATAGAGCTTTTGCCC
GFP-G228A	N683	G to C	CTATGAGTTCCGGAGAGCGATGAAAAGGTATGTG,
			CACATACCTTTTCATCGCTCTCCGGAACTCATAG
GFP-S250A	N748	T to G	CCTCTGCGCCGCCAGTAACACCGAG,
			CTCGGTGTTACTGGCGGCGCAGAGG
GFP-S251A	N751-752	AG to GC	CTCTGCGCCTCCGCTAACACCGAGACG,
			CGTCTCGGTGTTAGCGGAGGCGCAGAG
GFP-T255A	N763	A to G	CCAGTAACACCGAGGCGGTACACATCTTC,
			GAAGATGTGTACCGCCTCGGTGTTACTGG
GFP-H257A	N769-770	CA to GC	CACCGAGACGGTAGCCATCTTCAAGCTGGAAC,
			GTTCCAGCTTGAAGATGGCTACCGTCTCGGTG
GFP-S335A	N1003-1004	AG to GC	GCTAGTTGCGTCATCCGCTGGACACCTTTATATG,
			CATATAAAGGTGTCCAGCGGATGACGCAACTAGC
GFP-G336A	N1007	G to C	CTAGTTGCGTCATCCAGTGCACACCTTTATATG,
			CATATAAAGGTGTGCACTGGATGACGCAACTAG

**Figure 2:**
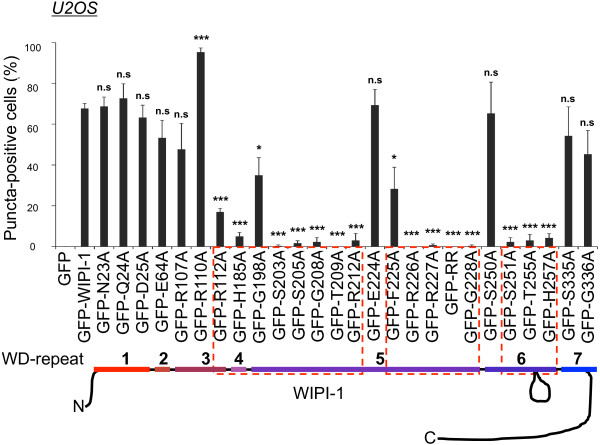
**Quantitative puncta-formation analysis of GFP-tagged wild-type or mutant WIPI-1 variants in U2OS cells.** U2OS cells transiently expressing GFP-WIPI-1 or GFP-tagged mutant WIPI-1 variants were treated for 3 h with control medium (CM), 233 nM wortmannin (WM), 300 nM rapamycin (RM), or rapamycin plus wortmannin (RM/WM), fixed and analyzed by confocal laser-scanning microscopy. Representative images and quantifications from all treatments are provided (Additional file 3: Table S1, Additional file 4: Figure S3, Additional file 5: Figure S4). From this, the quantification of rapamycin-treated cells is presented here as the percentage of puncta-positive cells for wild-type and mutant GFP-WIPI-1 variants (300 cells per condition, n = 3). P-values (reference GFP-WIPI-1): n.s. ≥ 0.05, * < 0.05, *** < 0.001.

We initiated the characterization of the generated mutants by conducting quantitative, fluorescence-based GFP-WIPI-1 puncta-formation analysis [37,38] upon transient expression (48 h) of the GFP control, wild-type or either of the generated GFP-WIPI-1 mutant in human U2OS cells (Figure 1B) followed by the following 3 h treatments. Application of control medium (CM) was used to score for autophagosomal membrane localization of GFP-WIPI-1 in cells undergoing basal autophagy. Rapamycin (RM) was applied to inhibit mTORC1 hence to induce autophagy. Wortmannin (WM) was used to either inhibit basal autophagy, or in combination with rapamycin (RM/WM) to counteract the induction of autophagy. Rapamycin-mediated autophagy was controlled by LC3-lipidation assays (Additional file 2: Figure S2). The number of puncta-positive cells was quantified (n = 3) from 300 individual cells for each condition (Additional file 3: Table S1; Additional file 4: Figure S3) and representative confocal images are presented (Additional file 5: Figure S4). From this, the quantification of puncta-positive cells upon RM administration along with p-value calculations is provided in Figure 2. With regard to wild-type GFP-WIPI-1, RM administration resulted in a significant reduction in the number of puncta-positive cells when the following mutants were expressed: R112A, H185A, G198A, S203A, S205A, G208A, T209A, R212A, F225A, R226A, R227A, G228A, S251A, T255A, H257A. Within this group, the following mutants were unable to form puncta: S203A, S205A, G208A, T209A, R212A, R226A, R227A, G228A, S251A, T255A, H257A. Exceptional, the expression of the R110 mutant resulted in an increased number of puncta-positive cells. The expression of the following mutants did not result in a significant alteration in puncta formation with regard to wild-type GFP-WIPI-1: N23A, Q24A, D25A, E64A, R107A, E224A, S250A, S335A, G336A (Figure 2, Additional file 3: Table S1, Additional file 4: Figure S3).

**Figure 3:**
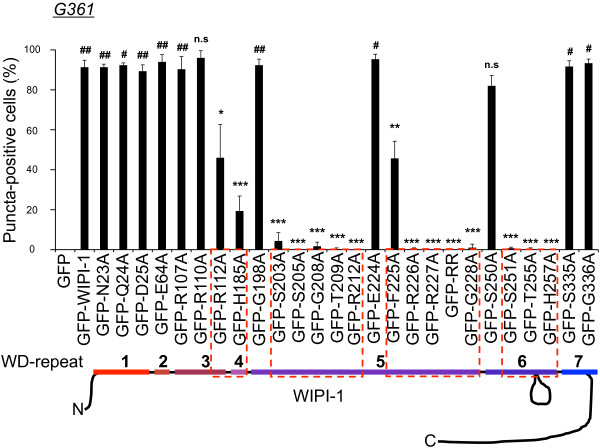
**Quantitative puncta-formation analysis of GFP-tagged wild-type or mutant WIPI-1 variants in G361 cells.** G361 cells transiently expressing GFP-WIPI-1 or GFP-tagged mutant WIPI-1 variants were treated for 3 h with 300 nM rapamycin (RM), fixed and analyzed by confocal laser-scanning microscopy. Quantifications (Additional file 6: Table S2) are presented as the percentage of puncta-positive cells for wild-type and mutant GFP-WIPI-1 variants (300 cells per condition, n = 3). P-values with regard to GFP-WIPI-1: n.s. ≥ 0.05, * < 0.05, ** < 0.01, *** < 0.001. *P*-values with regard to the corresponding condition in U2OS cells: # < 0.05, ## < 0.01.

We extended this analysis and transiently expressed wild-type GFP-WIPI-1 or either of the mutants in human G361 cells that also express high levels of endogenous WIPI-1. The quantification (n = 3) of GFP-WIPI-1 wild-type or mutant puncta-positive cells was performed upon RM administration in 300 individual cells (Additional file 6: Table S2) and p-value calculations conducted with regard to wild-type GFP-WIPI-1 (asterisks) or with regard to the number of puncta-positive cells achieved in U2OS cells (number sign) (Figure 3). Clearly, the expression of wild-type GFP-WIPI-1 or either of the puncta-formation competent mutants in G361 cells followed by rapamycin administration resulted in a significant increase in the number of puncta-positive G361 cells when compared to U2OS cells (Figure 3, number signs). In contrast to U2OS cells, the G198A mutant no longer showed a reduction in the number of puncta-positive cells when compared with wild-type GFP-WIPI-1 (Figure 3). All of the other mutants expressed in G361 cells (Figure 3) showed similar results when compared to U2OS cells (Figure 2).

**Figure 4:**
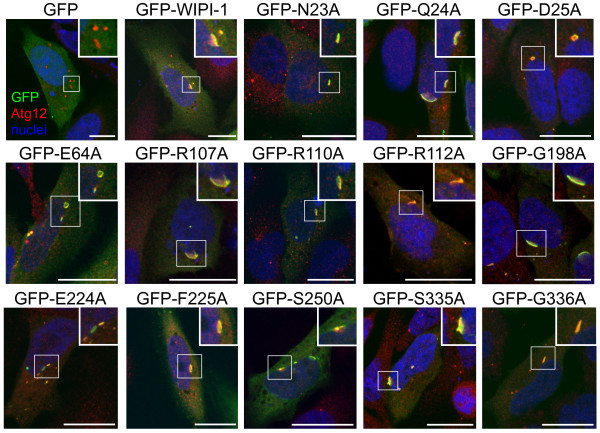
**Colocalization of endogenous Atg12 with wild-type GFP-WIPI-1 and puncta-formation competent GFP-WIPI-1 mutants upon rapamycin-mediated induction of autophagy.** U2OS cells transiently expressing GFP-WIPI-1 or GFP-tagged mutant WIPI-1 variants were treated for 3 h with 300 nM rapamycin, fixed, stained with anti-Atg12/Alexa 546 antibodies (red) and TOPRO3 (nuclei, blue), and analyzed by confocal laser-scanning microscopy. Merged images are shown. Scale bars 20 μM.

To detail the puncta analysis of mutant GFP-WIPI-1 proteins where the particular alanine substitution did not nullify the ability to localize at autophagosomal membranes (fluorescent puncta), we visualized this group of mutants (N23A, Q24A, D25A, E64A, R107A, R110A, R112A, G198A, E224A, F225A, S250A, S335A, G336A) or wild-type GFP-WIPI-1 (green) along with endogenous Atg12 (red) upon rapamycin-induced autophagy. Representative confocal images are presented, demonstrating that wild-type GFP-WIPI-1 as well as the mutants capable of puncta-formation co-localized (yellow) with Atg12 at perinuclear structures, reflecting ER-associated phagophore membranes (Figure 4). WIPI-1 mutants found to be incompetent for PtdIns(3)P binding were distributed throughout the cytoplasm and did not co-localize with formed Atg12 puncta upon rapamycin-mediated mTORC1 inhibition (data not shown).

**Figure 5:**
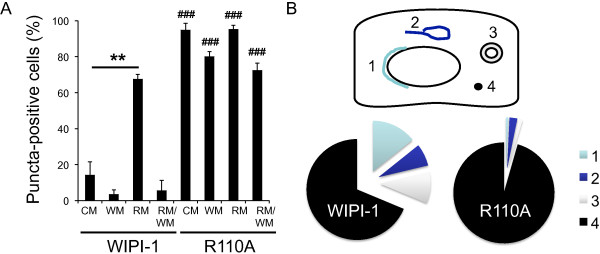
**Puncta analysis of the R110A mutant. (A)** P-value calculation for quantitative R110A puncta-formation analysis (Additional file 3: Table S1, Additional file 4: Figure S3, Additional file 5: Figure S4) with regard to CM: ** < 0.01; with regard to wild-type GFP-WIPI-1: ### < 0.001. **(B)** 50 puncta structures of GFP-WIPI-1 or GFP-R110A were categorized (1–4). Further supplementary material is available (Additional file 7: Figure S5).

During quantitative confocal microscopy it became apparent that the R110A puncta structures differed from wild-type GFP-WIPI-1 puncta. The R110A mutant showed a significant increase in the number of puncta-positive cells in all of the treatments (CM, WM, RM, RM/WM) when compared to wild-type GFP-WIPI-1 in U2OS cells. In addition, wortmannin did not abolish the ability to form distinct intracellular puncta (Figure 5A, Additional file 3: Table S1, Additional file 4: Figure S3). In order to compare the R110A puncta structures with wild-type GFP-WIPI-1, we distinguished between four categories of characteristic cytoplasmic WIPI-1 puncta appearing upon the induction of autophagy [29,39]: large elongated perinuclear structures (category 1), large lasso-like structures (category 2), large circular structures/vesicles (category 3), small dots (category 4) (Figure 5B). According to these four categories we quantified the puncta structures in GFP-WIPI-1 wild-type and R110A cells upon rapamycin administration. Predominantly, R110A puncta appeared as small cytoplasmic dots of category 4 (95.57%) and only a minority of puncta (4.43%) as large puncta of categories 1–3. In contrast, 31.295% of wild-type GFP-WIPI-1 puncta-positive cells showed large structures (categories 1–3) and 68.705% small cytoplasmic dots (category 4) (Figure 5B, Additional file 7: Figure S5). By applying this puncta analysis to further alanine mutants of conserved amino acids that cluster across the top of the propeller blades 1–3 in WIPI-1, we also found that a decrease of large puncta formation was apparent when D24, E64, R107 and R112 mutants were expressed in U2OS cells (Additional file 7: Figure S5).

**Figure 6:**
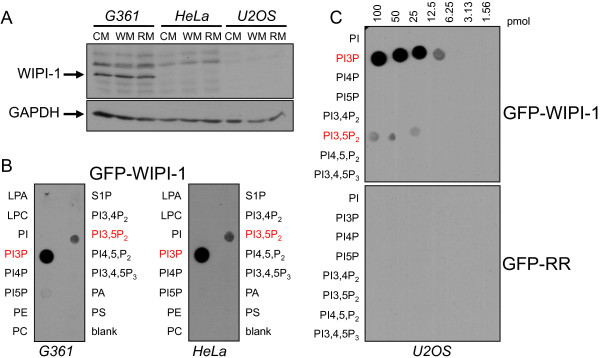
**GFP-WIPI-1 binding to PtdIns(3)P and PtdIns(3,5)P**_**2**_**. (A)** Western blot analysis of endogenous WIPI-1 protein in G361, HeLa or U2OS cells upon treatments with control medium (CM), 233 nM wortmannin (WM) or 300 nM rapamycin (RM). **(B)** Transient over-expression of GFP-WIPI-1 in G361 (left panel) or HeLa (right panel) cells followed by protein-phospholipid overlay assays using native cell extracts, adjusted to contain equivalent levels of GFP-WIPI-1 protein. **(C)** Protein-phospholipid overlay assay using native cell extracts from GFP-WIPI-1 or GFP-RR expressing U2OS cells.

To analyze the generated GFP-WIPI-1 mutants in their ability to bind PtdIns(3)P and PtdIns(3,5)P_2_, we first confirmed that over-expressed wild-type GFP-WIPI-1 in different cell lines that either express detectable (G361) or undetectable (HeLa, U2OS) levels of endogenous WIPI-1 protein (Figure 6A), predominantly binds PtdIns(3)P and to a minor extend PtdIns(3,5)P_2_ (Figure 6B and 6C upper panel). As expected, the RR mutant (R226A/R227A) did not bind to either of the phospholipids (Figure 6C lower panel).

**Figure 7:**
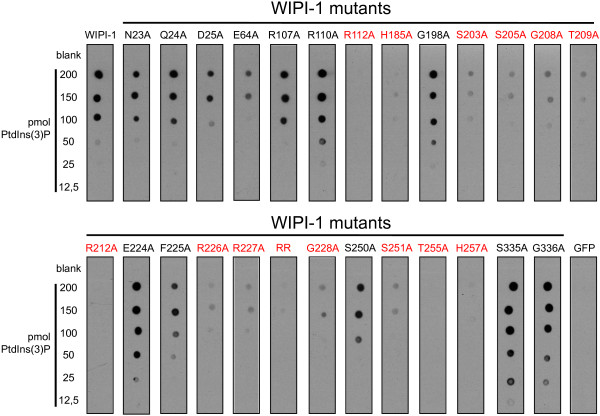
**Protein-phospholipid overlay assays with wild-type and mutant GFP-WIPI-1 variants.** From U2OS cells transiently expressing GFP-WIPI-1 or GFP-tagged mutant WIPI-1 variants native cell extracts were generated in parallel and used to overlay membrane-immobilized PtdIns(3)P (12,5–200 pmol) followed by anti-GFP ECL detection. Prior to overlaying the membranes with native cell extracts, the volumes of the different extracts were adjusted to include equivalent levels of GFP-WIPI-1 wild-type or mutant protein, judged by anti-GFP western blotting (not shown). Representative results are shown (n = 3). In red, GFP-tagged WIPI-1 mutants unable to bind to PtdIns(3)P.

**Figure 8:**
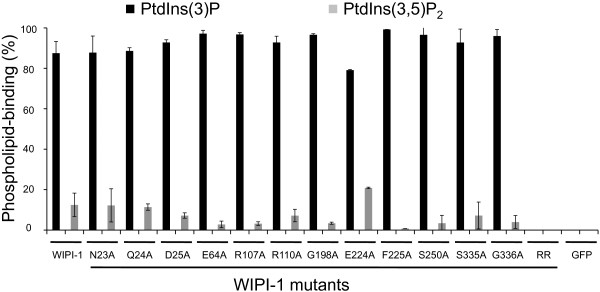
**GFP-WIPI-1 mutants capable of PtdIns(3)P can also bind to PtdIns(3,5)P**_**2**_**.** Quantification of phospholipid-protein overlay assays using membrane-immobilized PtdIns(3)P and PtdIns(3,5)P_2_, and native cell extracts from U2OS cells transiently expressing GFP-tagged wild-type or mutant WIPI-1 variants (n = 2). Total intensities of anti-GFP ECL signals at PtdIns(3)P and PtdIns(3,5)P_2_ positions were set to 100% for each variant and the very approximate percentage of PtdIns(3)P and PtdIns(3,5)P_2_ binding calculated.

By using different concentrations of immobilized PtdIns(3)P (12.5–200 pmol), protein-phospholipid overlay assays (n = 3) were conducted with transiently over-expressed GFP-WIPI-1 mutants along with wild-type GFP-WIPI-1 as a positive and GFP as a negative control from native U2OS cell extracts (Figure 7). Of note, equal GFP-WIPI-1 wild-type or mutant protein levels in native cell extracts used for protein-phospholipid overlay assays were adjusted upon western blot analysis (data not shown). Likewise, the mutants unable to form puncta (Figure 2, Figure 3) were unable to bind PtdIns(3)P (Figure 7, highlighted in red). In addition, the R112A and H185A mutants with reduced puncta-formation ability were also unable to bind PtdIns(3)P (Figure 7, highlighted in red). Further, mutants unable to bind to PtdIns(3)P did also not bind to PtdIns(3,5)P_2_ (data not shown). From the group of mutants able to bind PtdIns(3)P and PtdIns(3,5)P_2_, we addressed the very approximate PtdIns(3)P: PtdIns(3,5)P_2_ binding ratio. Using GFP-WIPI-1 as a positive and both the RR mutant and GFP as negative controls, we conducted protein-phospholipid overlay assays with immobilized PtdIns(3)P and PtdIns(3,5)P_2_ on the same membrane (data not shown). Based on anti-GFP ECL densitometry we calculated the approximate percentage of bound GFP-WIPI-1 to either of the phospholipids (Figure 8, n = 2). This result indicated that the mutants tested did not show key differences in their approximate PtdIns(3)P: PtdIns(3,5)P_2_ binding ability when compared to wild-type GFP-WIPI-1 (Figure 8).

**Figure 9:**
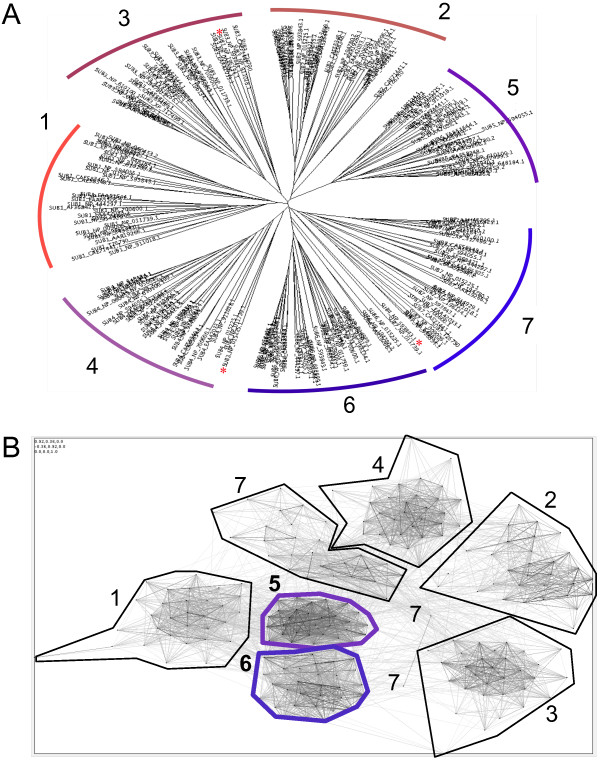
**Bioinformatic analysis of WIPI propeller blades. (A)** Phylogenetic and **(B)** cluster analysis of individual (1–7) beta-propeller blade sequences of the WIPI protein family [29].

The results achieved from characterizing the generated mutant GFP-WIPI-1 proteins are summarized in Table 2. From this analysis it became apparent that the evolutionarily conserved residues located in propeller blades 5 and 6 should fold into a 3-dimensional motif to confer direct PtdIns binding. Of note, propeller blade 5 and 6 sequences represent the most homologous protein region within the WIPI protein family, as shown by bioinformatic cluster analysis (Figure 9).

**Table 2:** Characterization of WIPI-1 mutants

**WIPI-1**	**Puncta**	**PtdIns(3)P**	**PtdIns(3,5)P**_**2**_	**Atg12**
**Mutant**	**formation**	**binding**	**binding**	**colocalization**
GFP-N23A	+	+	+	+
GFP-Q24A	+	+	+	+
GFP-D25A	+	+	+	+
GFP-E64A	+	+	+	+
GFP-R107A	+	+	+	+
GFP-R110A	+	+	+	+
GFP-R112A	+	-	n/a	+
GFP-H185A	+	-	n/a	n/a
GFP-G198A	+	+	+	+
GFP-S203A	-	-	-	-
GFP-S205A	-	-	-	-
GFP-G208A	-	-	-	-
GFP-T209A	-	-	-	-
GFP-R212A	-	-	-	-
GFP-E224A	+	+	+	+
GFP-F225A	+	+	+	+
GFP-R226A	-	-	-	-
GFP-R227A	-	-	-	-
GFP-RR	-	-	-	-
GFP-G228A	-	-	-	-
GFP-S250A	+	+	+	+
GFP-S251A	-	-	-	-
GFP-T255A	-	-	-	-
GFP-H257A	-	-	-	-
GFP-S335A	+	+	+	+
GFP-G336A	+	+	+	+

**Figure 10:**
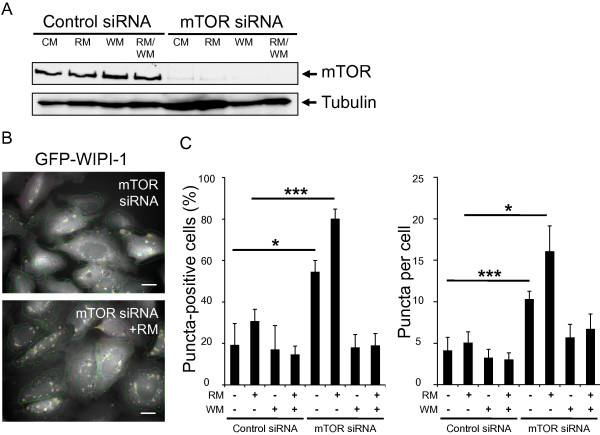
**Downregulation of mTOR elevates the number of cells that display GFP-WIPI-1 puncta. (A)** Human U2OS cells stably expressing GFP-WIPI-1 were transfected with 20 nM control siRNA or siRNA targeting mTOR. 48 h post-transfection the cells were treated with control medium (CM), rapamycin (RM), wortmannin (WM) or rapamycin plus wortmannin (RM/WM) for 2 h, and a representative western blot analysis from 3 independent experiments confirmed mTOR down regulation. **(B)** Fluorescence images were automatically acquired and **(C)** analyzed, and results (600–800 cells per condition, n = 3) expressed as GFP-WIPI-1 puncta-positive cells (left panel) or GFP-WIPI-1 puncta per cell (right panel). P-values: * < 0.05, *** < 0.001. Scale bars 20 μM.

As we employed rapamycin-mediated mTORC1 inhibition to induce autophagy for the characterization of the generated WIPI-1 mutants, we functionally addressed if siRNA-mediated downregulation of mTOR (Figure 10A) would likewise result in an increase of WIPI-1 puncta-positive cells. For this aim we used our previously established automated fluorescent puncta-image acquisition (Figure 10B) and analysis (Figure 10C) platform upon siRNA transfections of stable GFP-WIPI-1 U2OS cells. Clearly, down regulation of mTOR resulted in a significant increase of both GFP-WIPI-1 puncta-positive cells (Figure 10C, left panel) and puncta per individual cell (Figure 10C, right panel). In the presence of RM these levels further increased, and decreased in the presence of WM (Figure 10C).

**Figure 11:**
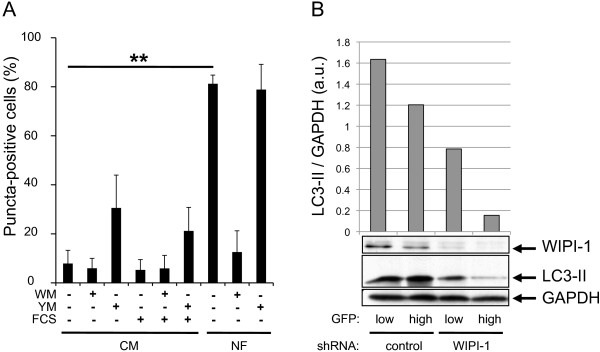
**PIKfyve inhibition elevates the number of cells that display WIPI-1 puncta, and WIPI-1 downregulation decreases LC3 lipidation. (A)** Human G361 cells were treated for 3 h either with YM201636 (YM) or wortmannin (WM) diluted in complete control medium (CM) with or without serum (FCS) or in serum- and amino acid-free medium (NF), followed by immunostaining of endogenous WIPI-1 using anti-WIPI-1/Alexa 488 antibodies and quantitative fluorescence microscopy. Results are expressed as the number of WIPI-1 puncta-positive cells (150 cells per condition, n = 3). P-value: ** < 0.01. **(B)** G361 cells were transfected with GFP-labelled control shRNA or shRNA targeting WIPI-1, and GFP-expressing cells were sorted into two groups with regard to high or low GFP intensities. Anti-WIPI-1 and anti-LC3 ECL analysis was conducted and signal intensities normalized over GAPDH. Representative results are shown (n = 2).

To confirm that the function of WIPI-1 at the onset of autophagy reflects its binding to generated PtdIns(3)P rather than to PtdIns(3,5)P_2_, we employed the compound YM201636 (YM) to specifically block PtdIns(3,5)P_2_ production by PIKfyve-mediated phosphorylation of PtdIns(3)P [40]. YM was added to control medium (CM), FCS-free CM or nutrient-free medium (NF) lacking both amino acids and FCS. WM was employed in parallel to inhibit PtdIns(3)P generation. Endogenous WIPI-1 was visualized by indirect immunofluorescence and the number of WIPI-1 puncta-positive cells was determined from a total of 150 individual cells for each condition (n = 3) (Figure 11A). In CM, the addition of YM resulted in an increase of WIPI-1 puncta-positive cells, indicating that indeed, PtdIns(3)P is bound by WIPI-1 at autophagosomal membranes. Further, by analyzing shRNA-mediated down regulation of WIPI-1 in G361 we confirmed that the PtdIns(3)P-effector function of WIPI-1 is essential for LC3 lipidation at the onset of autophagy (Figure 11B) [32].

## Discussion

**Figure 12:**
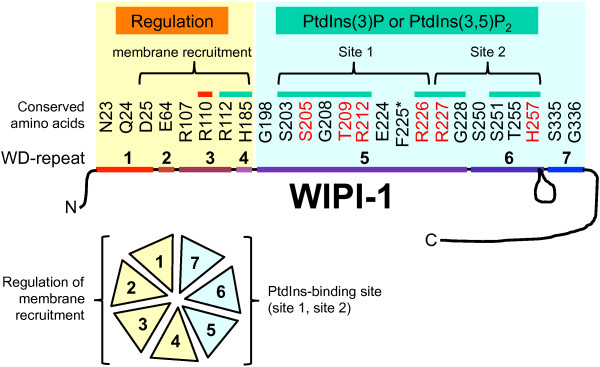
**Graphical interpretation of the results achieved from the analysis of generated WIPI-1 mutants.** See discussion for details.

**Figure 13:**
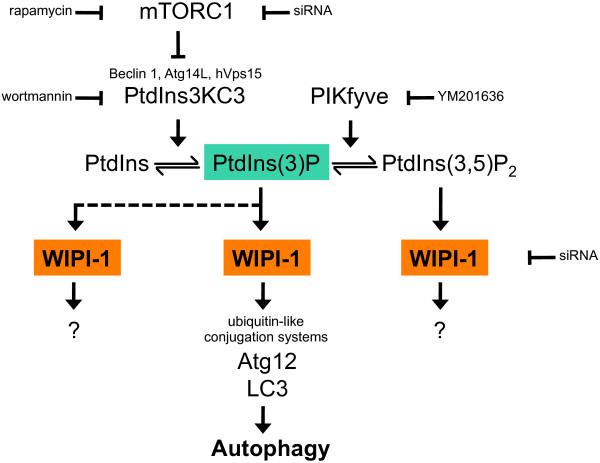
**A model for the role of WIPI-1 as a PtdIns(3)P effector at the onset of autophagy in human tumour cells.** As indicated, different compounds and siRNA's have been used in this study. See discussion for details.

Using quantitative WIPI-1 puncta-formation analysis we functionally identified critical amino acids for PtdIns(3)P- mediated autophagosomal membrane binding of human WIPI-1 (Figure 12) downstream of mTORC1 inhibition and PtdIns3KC3 activation (Figure 13). We found that the residues S203, S205, G208, T209, R212, R226, R227, G228, S251, T255, H257 (Figure 12, highlighted with green bars), displaying a cluster across propeller blades 4–7 [29], are responsible for PtdIns(3)P-binding at autophagosomal membranes during auto-phagy initiation. In line, a subset of this group of residues (Figure 12, highlighted in red lettering) was recently identified to provide PtdIns-binding of HSV2, a yeast homolog of human WIPI-1 via two binding sites [30] (Figure 12, site 1, site 2). These critical residues are predominantly positioned on propeller blade 5 and 6 of human WIPI-1 (Figure 12), both of which we show to be the most homologous propeller blades throughout the WIPI protein family (Figure 9B). Because the WIPI propeller was differentiated into its seven blades at the time when both paralogous groups of the WIPI protein family evolved ( [29], Figure 9A), the ancestral function of WIPI proteins should be crucially defined by PtdIns-binding properties. Of note, puncta-formation and PtdIns(3)P-binding competent WIPI-1 mutants also bound to a minor extend to PtdIns(3,5)P_2_ as earlier found for wild-type WIPI-1 and WIPI-2 [24,36,37], demonstrating that identical amino acids confer binding to PtdIns(3)P or PtdIns(3,5)P_2_. The proposed binding of HSV2 to two phosphoinositides simultaneously (Figure 12, site 1, site 2) could lead to a simultaneous PtdIns(3)P/PtdIns(3,5)P_2_ binding of the WIPI propeller under certain circumstances. Hence phosphorylation of PtdIns(3)P to generate PtdIns(3,5)P_2_ could regulate the function of WIPI proteins as PtdIns effectors. However, since WIPI-1 puncta formation is elevated when PtdIns(3,5)P_2_ production is blocked (Figure 11A), the specific localization at autophagosomal membranes upon autophagy induction should indeed predominantly reflect PtdIns(3)P binding of WIPI-1.

In addition to the residues conferring PtdIns(3)P binding, two residues, R112 and H185, (Figure 12, highlighted with green bars) were unable to efficiently bind PtdIns(3) but to localize at Atg12-positive autophagosomal membranes, in particular when expressed in cells with high levels of endogenous WIPI-1. This strongly indicates that membrane recruitment is mediated by evolutionarily conserved protein-protein interactions that regulate specific membrane localization of WIPI proteins. Further, one particular residue, R110, might be responsible for the association of an as yet unidentified inhibitory factor, as membrane localization was independent of autophagy stimulation and insensitive to autophagy inhibition (Figure 12, highlighted with a red bar). From this we suggest that the amino acids of propeller blades 1–4 provide differential association sites for regulatory factors that confer specific membrane binding, and amino acids of propeller blades 5–7 direct PtdIns binding. In line, by investigating the co-localization of WIPI-1 and the FYVE domain (GFP-2xFYVE) that also binds PtdIns(3)P, we show that WIPI-1 and GFP-2xFYVE did not prominently colocalize at same membranes upon rapamycin-mediated autophagy (Additional file 8: Figure S6), further indicating that the specificity of WIPI-1 to localize at autophagosomal membranes should indeed be directed by regulatory interacting factors.

## Conclusions

Evolutionarily conserved residues in WIPI-1 functionally identified in this study should confer specific binding to i) PtdIns(3)P or PtdIns(3,5)P_2_ and to ii) conserved interacting factors that determine membrane recruitment and specificity (Figure 12). Our study further provides evidence that the PtdIns(3)P effector function of WIPI-1 is regulated downstream of mTORC1 inhibition and PtdIns3KC3 activation, and upstream of both Atg12 and LC3 conjugation systems (Figure 13). By binding to PtdIns(3)P or PtdIns(3,5)P_2_, we anticipate that WIPI-1 might also be involved in additional as yet unidentified functions (Figure 13).

## Methods

### Reagents

Rapamycin, wortmannin and YM201636 were obtained from Sigma-Aldrich; Bafilomycin A_1_ from Applichem; PIP strips, PIP arrays, D-*myo*-phosphatidylinositol-3-phosphate, D-*myo*-phosphatidylinositol-3,5-bisphosphate from Echelon Biosciences.

### Primary antibodies

Anti-GFP antibody was purchased from Roche, anti-Atg12L antiserum from Abgent, anti-LC3 antibody from Nanotools, anti-α-tubulin antibody from Sigma-Aldrich, anti-GAPDH antibody from HyTest, anti-mTOR from Cell Signaling. The rabbit polyclonal anti-WIPI-1 antiserum was described earlier [29].

### Secondary antibodies

Alexa Fluor 488 and Alexa Fluor 546 goat anti-mouse and anti-rabbit IgG antibodies were obtained from Invitrogen, anti-mouse IgG-HRP and anti-rabbit IgG-HRP antibodies from GE Healthcare.

### Fluorescent dyes

TO-PRO-3 was obtained from Invitrogen and DAPI was purchased from AppliChem.

### cDNA constructs

GFP-WIPI-1 (pEGFP.C1-WIPI-1α) [29] was used as DNA template for site-directed mutagenesis (Quick change II, Stratagene) to generate GFP-WIPI-1 mutants with synthesized (Purimex, Sigma-Aldrich) oligonucleotides (see Table 1). Construct integrities were verified by DNA sequencing.

### Cell culture and transfection

Human U2OS and G361 cells (both from ATCC) were cultured in DMEM, 10% FCS, 100 U/ml penicillin/100 μg/ml streptomycin, 5 mg/ml plasmocin (Invivogen) at 37°C, 5% CO_2_. The monoclonal U2OS cell line stably expressing GFP-WIPI-1 was described earlier [41] and cultured in the presence of 0.6 mg/ml G418 (Invitrogen). Cells were transfected with DNA using PromoFectin (Promocell) or Lipifectamine2000 (Invitrogen) according to the manufacturer's instruction. Using RNAiMax (Invitrogen) 20 nM control siRNA or siRNA targeting mTOR (both from Cell Signalling) were transfected according to the manufacturer's instruction for reverse transfection. Using Lipofectamine2000 G361 cells were transfected with shRNA constructs targeting human WIPI-1 and expressing GFP (SuperArray), followed by fluorescence-assisted cell sorting [42] of low or high GFP-expressing cells.

### Autophagy assays

Autophagy assays were conducted for 2 or 3 h using amino acid and serum-free medium (EBSS, from Sigma Aldrich), and by administration of rapamycin (300 or 500 nM), wortmannin (233 nM) or YM201636 (800 nM).

### Confocal laser-scanning microscopy

Cells were fixed (3.7% paraformaldehyde in PBS) and prepared for direct fluorescence of GFP-WIPI-1 or for indirect immunofluorescence of endogenous WIPI-1 or Atg12 using anti-WIPI-1 or anti-Atg12 antiserum at 1:25–1:50 and Alexa Fluor secondary antibody conjugates (Alexa 488 or Alexa 546) at 1:200–1:250. Z-stacks (10–20 optical sections of 0.5 μm) were acquired using Zeiss/Axiovert 100 M/LSM510 and a 63 × 1.4 DIC Plan-apochromat objective. Individual optical sections were used to analyse co-localization events. Projections of individual optical sections were used to generate final images.

### Automated high throughput GFP-WIPI-1 puncta-formation image acquisition and analyses

Stable U2OS GFP-WIPI-1 cells were cultured in 6 well plates, transfected with 20 nM siRNA's and 48 h post-transfection autophagy assays were performed for 2 h. Cells were fixed (3,7% PFA in PBS) and incubated with DAPI (5 μg/ml PBS). Using a high content platform (In Cell Analyzer 1000, GE Healthcare) equipped with a Nikon Plan Fluor ELWD 40 × 0,6 objective, automated GFP-WIPI-1 image acquisition and analysis (In Cell Analyzer Workstation 3.4) was conducted as previously described [41].

### Immunoblotting

Cells were lysed in hot Laemmli buffer and total protein extracts used for standard western blotting and ECL detection (GE Healthcare). Protein abundance was normalized over GAPDH by quantifying ECL signal intensities using the Personal Densitometer SI (Molecular Dynamics) and Image Quant 5.1.

### Phospholipid-protein overlay assay

Phosphatidylinositol-3-phosphate diC16 and Phosphatidylinositol-3,5-bisphosphate diC16 were solved in 1:2:0,8 CHCl_3_: MeOH: H_2_O and applied on a nitrocellulose membrane (Amersham Hybond^TM^-C Extra). Membrane-immobilized phospholipids were dried at room temperature in the dark for 1 h. Alternatively, PIP strips or PIP arrays (Echelon Biosciences) were used. As described earlier [37], membrane-immobilized phospholipids were rinsed in TBS and TBS/0.1% Tween, and blocked for 1 h at room temperature in TBS/0.1% Tween/3% BSA. Membranes were incubated with the soluble fractions (50 μg to 150 μg total protein) from native cell extracts (750 mM aminocaproic acid, 50 mM Bis-Tris, 0.5 mM EDTA, pH 7.0, Roche Protease inhibitor Cocktail) for 16 h at 4°C. Prior to this incubation step, aliquots of the soluble fractions were used for Bradford assays and to normalize protein expression by western blotting and densitometry. From this, native extract volumes were adjusted to contain equivalent levels of over-expressed proteins (wild-type GFP-WIPI-1 and the generated mutants). Finally, anti-GFP ECL detection was used to detect phospholipid-bound proteins.

### Statistical analysis

Mean values from 3 independent sets of experiments (± SD) were used to calculate p-values (heteroscedastic t-testing).

### Bioinformatics

Individual beta-propeller blade sequences of the WIPI protein family were analysed phylogenetically using CLANS cluster analysis [43] and ASATURA neighbor-joining phylogeny [44] as previously described [29].

## Abbreviations

AMPK: AMP-activated protein kinase; Atg: Autophagy related; DMEM: Dulbecco's modified eagle medium; EBSS: Earl's balanced salt solution; FIP200: Focal adhesion kinase family kinase interacting protein of 200 kDa; FYVE: Fab1p YOTB, Vac1p, EEA1; LC3: Microtubule-associated protein light chain 3; mTOR: Mammalian target of rapamycin; mTORC1: Mammalian target of rapamycin complex 1; PIKfyve: FYVE finger-containing phosphoinositide kinase; PtdIns(3)P: Phosphatidylinositol 3-phosphate; PtdIns3KC3: Phosphatidylinositol 3-kinase class III; PtdIns(3,5)P_2_: Phosphatidylinositol 3,5-bisphosphate; Ulk: UNC-51-like kinase; Vps: Vacuolar protein sorting; WIPI: WD-repeat protein interacting with phosphoinositides.

## Competing interests

The authors declare they have no competing interests.

## Authors' contributions

AG designed and conducted site-directed mutagenesis, characterized GFP-WIPI-1 mutants, analyzed the data and drafted the manuscript. DB carried out automated GFP-WIPI-1 puncta-formation analysis upon mTOR down regulation and analyzed the data. AH characterized the effect of YM201636 on WIPI-1 puncta formation and analyzed the data. TP-C conceived and designed the study, analyzed the data and wrote the manuscript. All authors read and approved the final manuscript.

## Supplementary Material

Additional file 1 **Figure S1.** Multiple amino acid sequence alignment of wild-type human WIPI-1 and generated mutants. Yellow: alanine substitutions of homologous residues within the WIPI protein family, red: alanine substitutions of invariant residues, green: alanine substitution of a nonhomologous residue. Click here for file

Additional file 2 **Figure S2.** LC3-lipidation analysis upon rapamycin-mediated autophagy in U2OS cells. Control U2OS cells or U2OS cells transiently expressing GFP-WIPI-1 were treated with rapamycin in the presence or absence of protease inhibitors (P.I.) followed by anti-LC3 western blot analysis from total protein extracts. The LC3-II/LC3-I ratio was determined by densitometry. Click here for file

Additional file 3 **Table S1.** Quantitative GFP-WIPI-1 puncta-formation analysis in U2OS cells. Treatments: control medium (CM), rapamycin (RM), rapamycin plus wortmannin (RM/WM), wortmannin (WM). The number of puncta-positive cells was determined in 100 cells per condition. The data from three independent experiments (dataset 1–3) along with mean values (%) is presented. Click here for file

Additional file 4 **Figure S3.** Graphical representation of the results provided in Additional file 3: Table S1 along with standard deviations. In addition, the number for non-puncta cells is also presented for each condition. In black: puncta-positive cells, in white: non-puncta cells. Click here for file

Additional file 5 **Figure S4.** Representative confocal images from the analysis provided in Additional file 3: Table S1. Bars: 20 μM. Click here for file

Additional file 6 **Table S2.** Quantitative GFP-WIPI-1 puncta-formation analysis in G361 cells. Treatment: rapamycin (RM). The number of puncta-positive cells was determined in 100 cells per dataset (1–3). Original data and mean values (%) are presented. Click here for file

Additional file 7 **Figure S5.** The percentage of small and large puncta structures displayed by wild-type and mutant GFP-WIPI-1 proteins upon rapamycin administration in U2OS cells. Images from Additional file 5: Figure S4 were used and 50 puncta structures were categorized for each GFP-WIPI-1 mutant. Click here for file

Additional file 8 **Figure S6.** Quantitative co-localization study of WIPI-1 and over-expressed GFP-2xFYVE. Using G361 cells transiently expressing GFP-2xFYVE, endogenous EEA1 or endogenous WIPI-1 was detected by indirect immunofluorescence. In addition, G361 cells transiently expressing both *myc*-tagged WIPI-1 and GFP-2xFYVE were subjected to anti-*myc* immunofluorescence. By confocal microscopy co-localization events (see arrows) were counted using 10 individual cells each. Endogenous as well as *myc*-tagged WIPI-1 co-localized with GFP-2xFYVE in 1 out of 10 cells (1 structure / cell). Bar: 20 μM. Click here for file
