# Supplementary material for: Defining regulatory and phosphoinositide-binding sites in the human WIPI-1 β-propeller responsible for autophagosomal membrane localization downstream of mTORC1 inhibition
Source: J Mol Signal. 2012 Oct 22;7:16. doi: 10.1186/1750-2187-7-16 (PMC3543385; doi:10.1186/1750-2187-7-16)
Supplement: Additional file 1 — Figure S1. Multiple amino acid sequence alignment of wild-type human WIPI-1 and generated mutants. Yellow: alanine substitutions of homologous residues within the WIPI protein family, red: alanine substitutions of invariant residues, green: alanine substitution of a nonhomologous residue. [file 1750-2187-7-16-S1.pdf]

Suppl. Figure S1

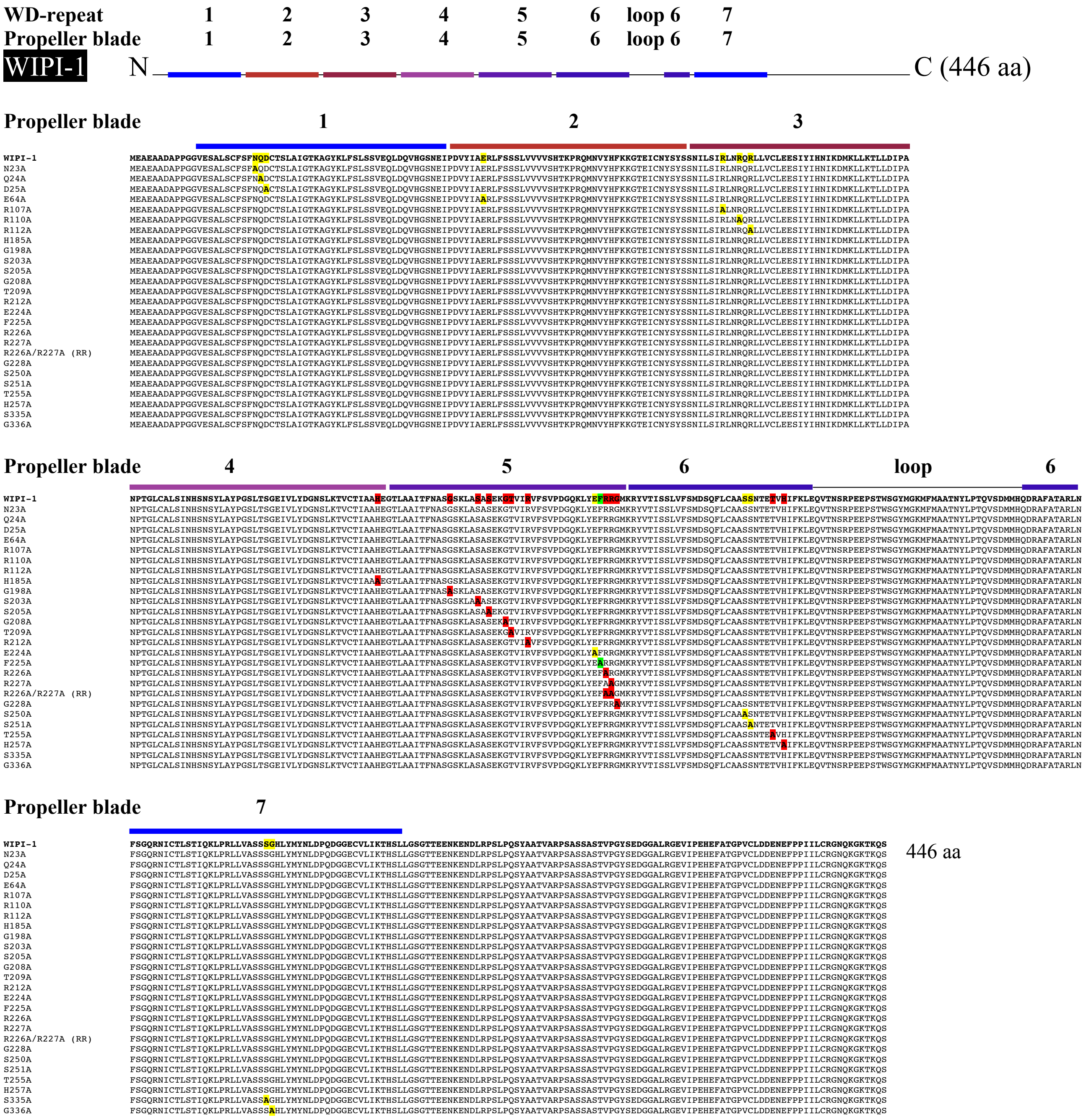

Alanin substitutions of homologous amino acids (in yellow)

Alanin substitutions of invariant amino (acids in red)

Alanin substitution of a non-homologous amino acid (in green)
