# Supplementary figures and images for: Defining regulatory and phosphoinositide-binding sites in the human WIPI-1 β-propeller responsible for autophagosomal membrane localization downstream of mTORC1 inhibition
Source: J Mol Signal. 2012 Oct 22;7:16. doi: 10.1186/1750-2187-7-16 (PMC3543385; doi:10.1186/1750-2187-7-16)

Suppl. Figure S2

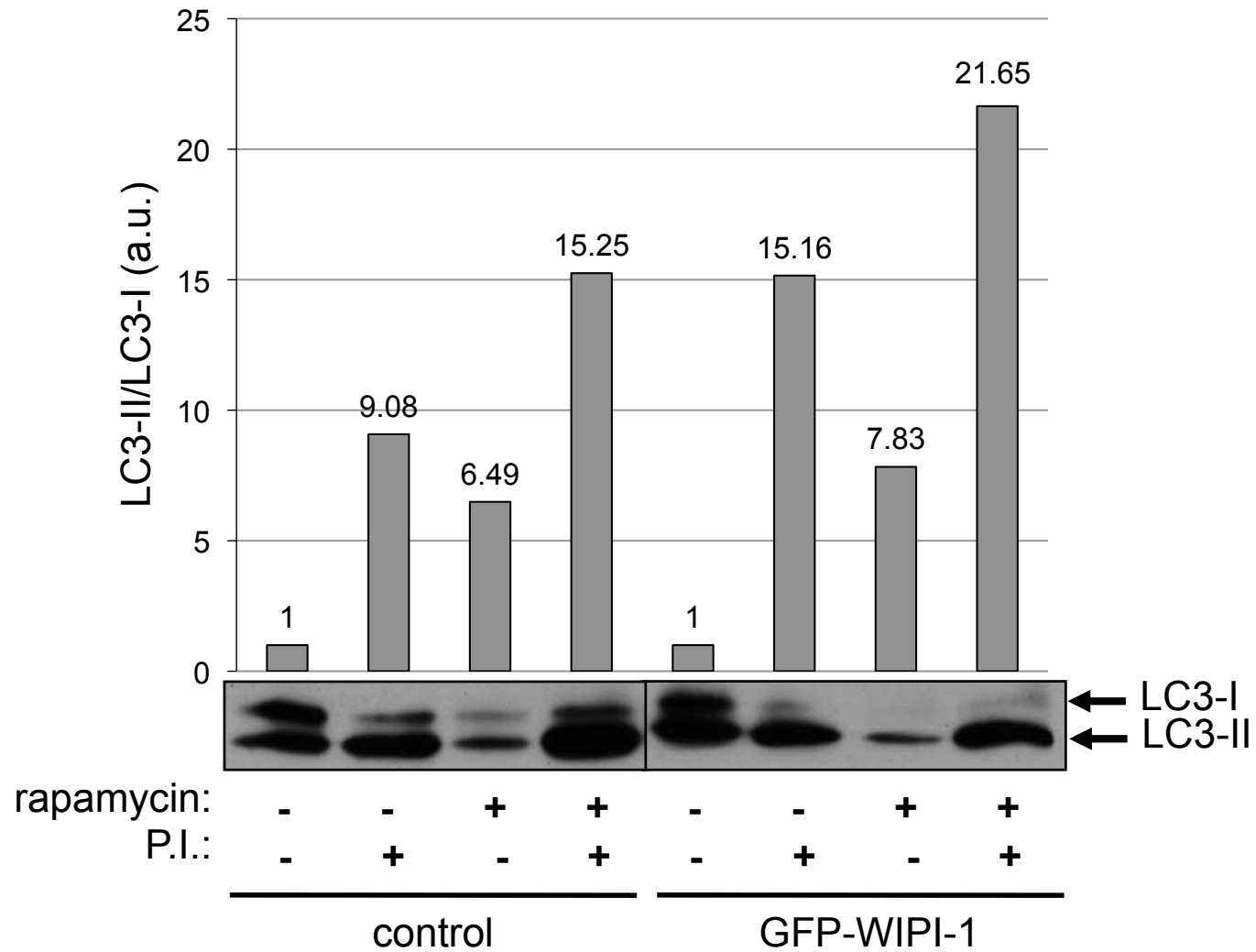

Supplement: Additional file 2 — Figure S2. LC3-lipidation analysis upon rapamycin-mediated autophagy in U2OS cells. Control U2OS cells or U2OS cells transiently expressing GFP-WIPI-1 were treated with rapamycin in the presence or absence of protease inhibitors (P.I.) followed by anti-LC3 western blot analysis from total protein extracts. The LC3-II/LC3-I ratio was determined by densitometry. [file 1750-2187-7-16-S2.pdf]

Suppl. Figure S3

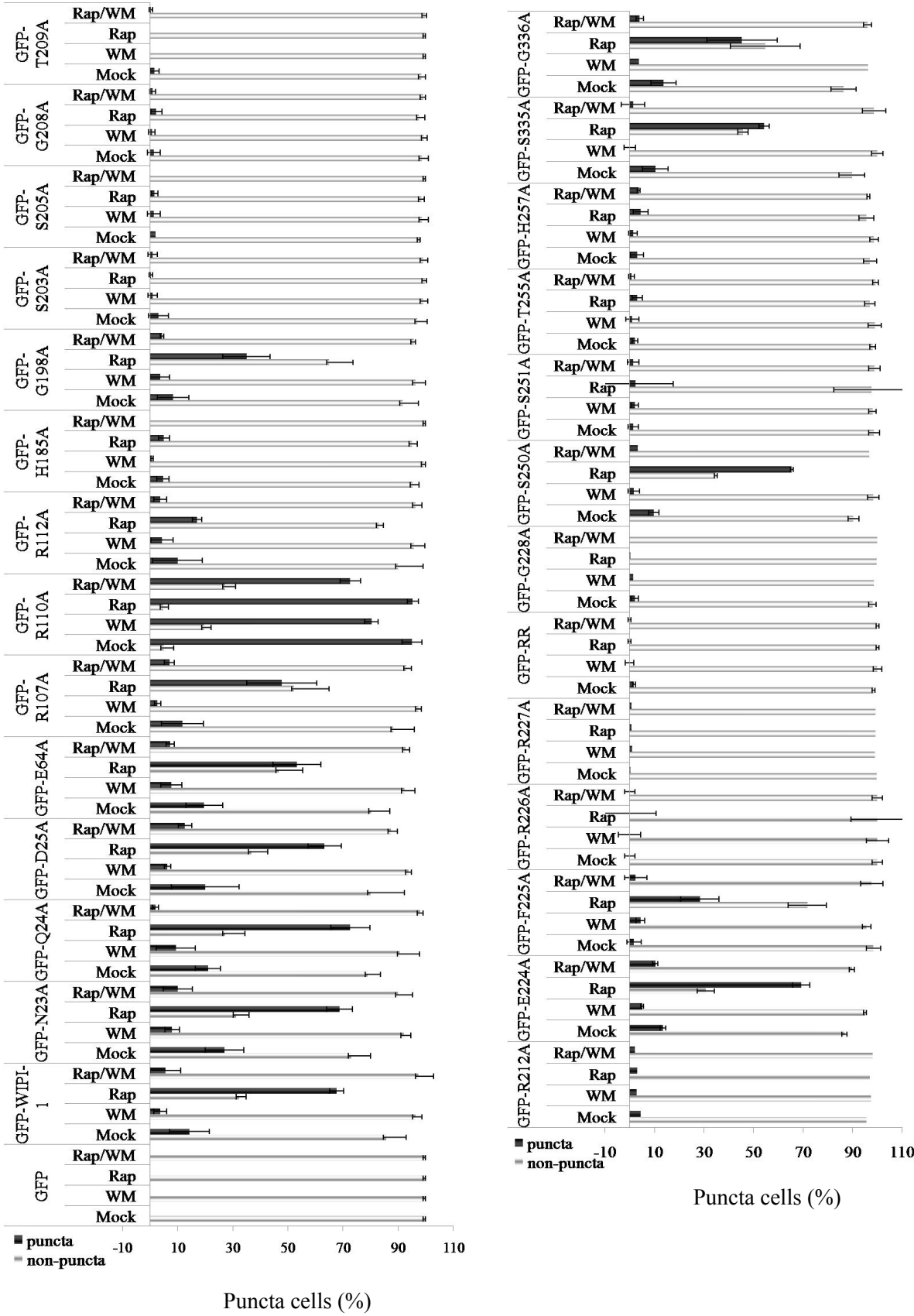

Supplement: Additional file 4 — Figure S3. Graphical representation of the results provided in Additional file 3: Table S1 along with standard deviations. In addition, the number for non-puncta cells is also presented for each condition. In black: puncta-positive cells, in white: non-puncta cells. [file 1750-2187-7-16-S4.pdf]

Suppl. Figure S4

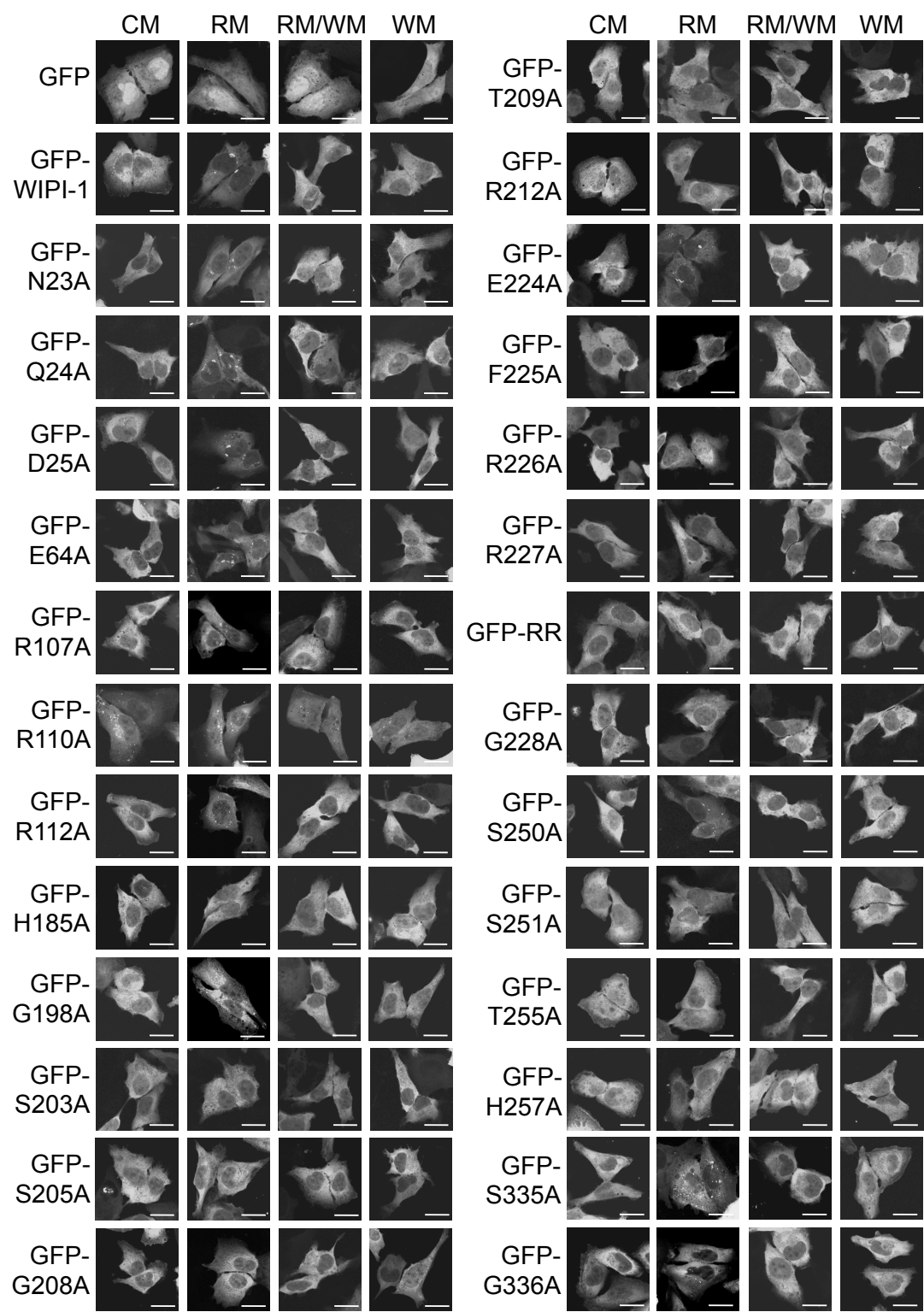

Supplement: Additional file 5 — Figure S4. Representative confocal images from the analysis provided in Additional file 3: Table S1. Bars: 20 μM. [file 1750-2187-7-16-S5.pdf]

Suppl. Figure S5

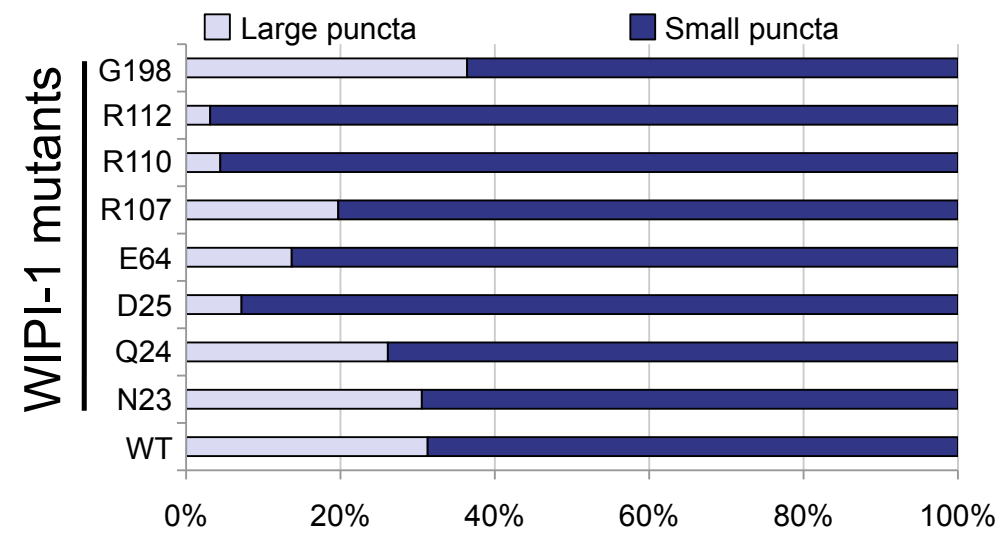

Supplement: Additional file 7 — Figure S5. The percentage of small and large puncta structures displayed by wild-type and mutant GFP-WIPI-1 proteins upon rapamycin administration in U2OS cells. Images from Additional file 5: Figure S4 were used and 50 puncta structures were categorized for each GFP-WIPI-1 mutant. [file 1750-2187-7-16-S7.pdf]

Suppl. Figure S6

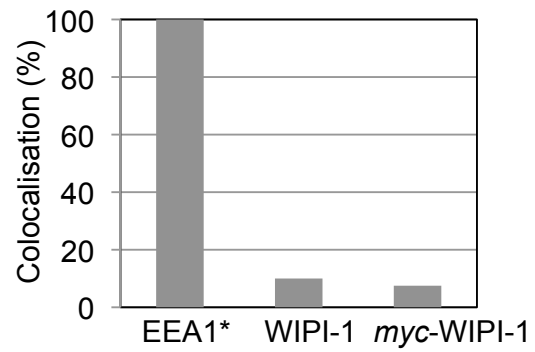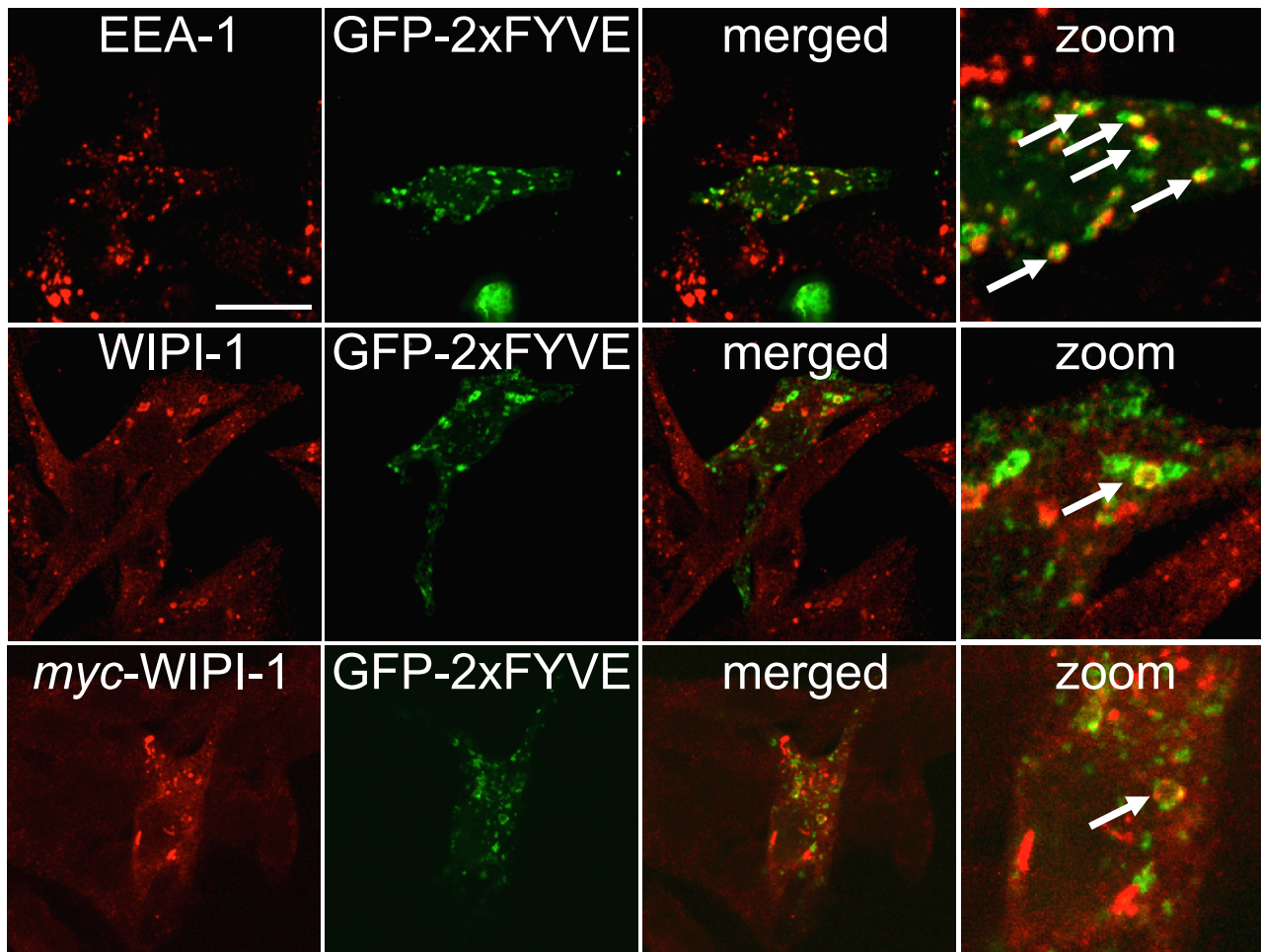

Supplement: Additional file 8 — Figure S6. Quantitative co-localization study of WIPI-1 and over-expressed GFP-2xFYVE. Using G361 cells transiently expressing GFP-2xFYVE, endogenous EEA1 or endogenous WIPI-1 was detected by indirect immunofluorescence. In addition, G361 cells transiently expressing both myc-tagged WIPI-1 and GFP-2xFYVE were subjected to anti-myc immunofluorescence. By confocal microscopy co-localization events (see arrows) were counted using 10 individual cells each. Endogenous as well as myc-tagged WIPI-1 co-localized with GFP-2xFYVE in 1 out of 10 cells (1 structure / cell). Bar: 20 μM. [file 1750-2187-7-16-S8.pdf]
