# Supplementary material for: Defining regulatory and phosphoinositide-binding sites in the human WIPI-1 β-propeller responsible for autophagosomal membrane localization downstream of mTORC1 inhibition
Source: J Mol Signal. 2012 Oct 22;7:16. doi: 10.1186/1750-2187-7-16 (PMC3543385; doi:10.1186/1750-2187-7-16)
Supplement: Additional file 3 — Table S1. Quantitative GFP-WIPI-1 puncta-formation analysis in U2OS cells. Treatments: control medium (CM), rapamycin (RM), rapamycin plus wortmannin (RM/WM), wortmannin (WM). The number of puncta-positive cells was determined in 100 cells per condition. The data from three independent experiments (dataset 1–3) along with mean values (%) is presented. [file 1750-2187-7-16-S3.pdf]

Table S1. Confocal microscopy of GFP-WIPI-1 puncta-positive human U2OS cells.

Dataset	1				2				3				Mean (%)			
	CM	RM	RM/WM	WM	CM	RM	RM/WM	WM	CM	RM	RM/WM	WM	CM	RM	RM/WM	WM
GFP	0	0	0	0	0	0	0	0	0	0	0	0	0.0	0.0	0.0	0.0
GFP-WIPI1	18	70	6	5	19	65	11	5	6	68	0	1	14.3	67.7	5.7	3.7
GFP-N23A	32	66	8	7	30	74	16	6	19	66	6	11	27.0	68.7	10.0	8.0
GFP-Q24A	20	79	2	10	26	65	3	2	17	74	1	16	21.0	72.7	2.0	9.3
GFP-D25A	34	69	10	5	15	64	15	7	11	57	13	7	20.0	63.3	12.7	6.3
GFP-E64A	23	55	6	12	12	61	9	5	24	44	7	6	19.7	53.3	7.3	7.7
GFP-R107A	5	50	5	4	10	59	8	2	20	34	8	2	11.7	47.7	7.0	2.7
GFP-R110A	91	96	77	78	98	97	70	83	96	93	71	80	95.0	95.3	72.7	80.3
GFP-R112A	1	16	1	5	10	19	5	0	19	16	5	8	10.0	17.0	3.7	4.3
GFP-H185A	2	5	0	1	6	7	0	0	6	3	0	1	4.7	5.0	0.0	0.7
GFP-G198A	15	34	4	4	5	44	4	0	5	27	5	7	8.3	35.0	4.3	3.7
GFP-S203A	0	0	0	0	2	0	0	0	7	1	3	3	3.0	0.3	1.0	1.0
GFP-S205A	2	3	0	0	2	1	0	4	2	1	0	0	2.0	1.7	0.0	1.3
GFP-G208A	4	4	1	0	0	3	2	2	0	0	0	0	1.3	2.3	1.0	0.7
GFP-T209A	2	0	1	0	0	0	0	0	3	0	0	0	1.7	0.0	0.3	0.0
GFP-R212A	5	7	3	2	3	1	1	3	5	1	2	3	4.3	3.0	2.0	2.7
GFP-E224A	10	67	15	3	15	63	6	6	15	78	10	6	13.3	69.3	10.3	5.0
GFP-F225A	1	17	4	9	4	30	0	4	0	38	3	0	1.7	28.3	2.3	4.3
GFP-R226A	0	0	0	0	0	0	0	0	0	0	0	0	0.0	0.0	0.0	0.0
GFP-R227A	1	1	0	3	0	1	1	0	0	0	1	0	0.3	0.7	0.7	1.0
GFP-RR	3	0	0	0	2	0	0	0	0	0	0	0	1.7	0.0	0.0	0.0
GFP-G228A	4	1	0	4	1	0	0	0	1	0	0	0	2.0	0.3	0.0	1.3
GFP-S250A	9	71	2	3	8	77	6	2	12	48	2	0	9.7	65.3	3.3	1.7
GFP-S251A	0	4	0	0	2	0	2	5	2	3	2	1	1.3	2.3	1.3	2.0
GFP-T255A	5	3	1	3	0	0	0	0	1	6	1	0	2.0	3.0	0.7	1.0
GFP-H257A	9	5	9	4	0	2	2	0	0	6	0	0	3.0	4.3	3.7	1.3
GFP-S335A	15	63	0	0	5	38	1	0	11	62	3	0	10.3	54.3	1.3	0.0
GFP-G336A	18	56	2	6	8	33	7	5	15	47	3	0	13.7	45.3	4.0	3.7

Control medium (CM), rapamycin (RM), wortmannin (WM), rapamycin plus wortmannin (RM/WM).
