# Supplementary material for: Defining regulatory and phosphoinositide-binding sites in the human WIPI-1 β-propeller responsible for autophagosomal membrane localization downstream of mTORC1 inhibition
Source: J Mol Signal. 2012 Oct 22;7:16. doi: 10.1186/1750-2187-7-16 (PMC3543385; doi:10.1186/1750-2187-7-16)
Supplement: Additional file 6 — Table S2. Quantitative GFP-WIPI-1 puncta-formation analysis in G361 cells. Treatment: rapamycin (RM). The number of puncta-positive cells was determined in 100 cells per dataset (1–3). Original data and mean values (%) are presented. [file 1750-2187-7-16-S6.pdf]

Table S2. Confocal microscopy of GFP-WIPI-1 puncta-positive human G361 cells.

Dataset	1 (RM)	2 (RM)	3 (RM)	Mean (%)
GFP	0	0	0	0.0
GFP-WIPI1	88	91	95	91.3
GFP-N23A	93	90	91	91.3
GFP-Q24A	93	93	91	92.3
GFP-D25A	88	87	93	89.3
GFP-E64A	95	90	97	94.0
GFP-R107A	94	83	94	90.3
GFP-R110A	99	97	92	96.0
GFP-R112A	65	39	34	46.0
GFP-H185	27	12	19	19.3
GFP-G198A	95	89	93	92.3
GFP-S203A	9	1	3	4.3
GFP-S205A	0	0	0	0.0
GFP-G208A	4	1	0	1.7
GFP-T209A	0	0	1	0.3
GFP-R212A	0	0	0	0.0
GFP-E224A	98	95	93	95.3
GFP-F225A	55	38	44	45.7
GFP-R226A	0	0	1	0.3
GFP-R227A	0	0	0	0.0
GFP-RR	0	0	0	0.0
GFP-G228A	0	0	3	1.0
GFP-S250A	88	79	79	82.0
GFP-S251A	0	1	1	0.7
GFP-T255A	0	0	1	0.3
GFP-H257A	0	0	0	0.0
GFP-S335A	90	95	90	91.7
GFP-G336A	95	91	94	93.3

Rapamycin (RM).
